# Large-scale implementation of alcohol brief interventions in new settings in Scotland: a qualitative interview study of a national programme

**DOI:** 10.1186/s12889-015-1527-6

**Published:** 2015-03-25

**Authors:** Niamh Fitzgerald, Lucy Platt, Susie Heywood, Jim McCambridge

**Affiliations:** Institute for Social Marketing, UK Centre for Tobacco and Alcohol Studies, University of Stirling, Stirling, FK9 4LA, Scotland UK; Institute for Health and Wellbeing Research, Robert Gordon University, Riverside Campus, Aberdeen, AB10 7GJ UK; Faculty of Public Health and Policy, London School of Hygiene and Tropical Medicine, 15-17 Tavistock Place, London, WC1H 9SH UK; Glasgow City Community Health Partnership, North-East Sector, Eastbank Health Promotion & Training Centre, Academy Street, Glasgow, G32 9AA UK

**Keywords:** Alcohol, Brief intervention, Implementation, Brief advice, Target

## Abstract

**Background:**

This study aimed to explore experiences of implementation of alcohol brief interventions (ABIs) in settings outside of primary healthcare in the Scottish national programme. The focus of the study was on strategies and learning to support ABI implementation in settings outside of primary healthcare in general, rather on issues specific to any single setting.

**Methods:**

14 semi-structured telephone interviews were conducted with senior implementation leaders in antenatal, accident and emergency and wider settings and audio-recorded. Interviews were analysed inductively.

**Results:**

The process of achieving large-scale, routine implementation of ABI proved challenging for all involved across the settings. Interviewees reported their experiences and identified five main strategies as helpful for strategic implementation efforts in any setting: (1) Having a high-profile target for the number of ABIs delivered in a specific time period with clarity about whose responsibility it was to implement the target; (2) Gaining support from senior staff from the start; (3) Adapting the intervention, using a pragmatic, collaborative approach, to fit with current practice; (4) Establishing practical and robust recording, monitoring and reporting systems for intervention delivery, prior to widespread implementation; and (5) Establishing close working relationships with frontline staff including flexible approaches to training and readily available support.

**Conclusions:**

This qualitative study suggests that even with significant national support, funding and a specific delivery target, ABI implementation in new settings is not straightforward. Those responsible for planning similar initiatives should critically consider the relevance and value of the five implementation strategies identified.

## Background

Alcohol brief interventions (ABIs) are heterogeneous interventions [1-4] that include ‘short conversations aiming in a non-confrontational way to motivate individuals to think about and/or plan a change in their drinking behaviour in order to reduce their consumption and/or their risk of harm’ [5]. ABIs have historically included the use of a screening questionnaire to explore an individual’s consumption level and risk of alcohol problems, and the provision of personalised feedback based on such screening [6].

Systematic reviews have concluded that ABI delivery in primary care has modest efficacy in reducing alcohol consumption in hazardous and harmful drinkers [7,8]. Evidence for efficacy in Accident and Emergency (A&E) [9,10], general hospital [11], antenatal [12,13] and other settings including education, pharmacy and criminal justice [14-16] is less convincing. Furthermore the appropriateness, targeting and timing of screening for alcohol problems within various settings have been the subject of debate [17-21]. Notwithstanding this, implementation of ABIs has been recommended in the UK in a wide range of such settings (National Institute for Health and Care Excellence [22]; Scottish Government [23]). The Scottish Government national alcohol strategy states “we know brief interventions are effective in helping people to reduce their drinking, and as such their risk of alcohol related harm” in describing the national ABI programme, which prioritises implementation in A&E, antenatal and primary care settings [24].

The implementation of interventions novel to practice in health and other settings is not straightforward [25,26]. One review identified four strategies for implementing ABIs in primary care, all aimed at practitioners, and concluded that material utilisation, screening, and brief intervention rates “*increased with the intensity of the intervention effort, i.e. the amount of training and/or support provided*” [27]. Another found that “*adequate resources, training and the identification of those at risk without stereotyping”* were the main facilitators of implementation but concluded that further research was needed outside of primary care [28]. Implementation issues in other settings are likely to both share similarities with primary care, and also be distinct.

From April 2008, a Scottish Government target [29] required the NHS as a whole to deliver a minimum number of ABIs in the three priority settings (primary care, A&E, antenatal) and later a range of other ‘wider’ settings [5,23]. The national target was divided up into targets for each local health service (‘health board’) in Scotland, each of which was required to report regularly on implementation progress. The national initiative was well-resourced [24], encouraged local ownership of implementation [29,30], focused on addressing risky drinking rather than alcohol dependence [30,31], emphasised professional education for nurses and doctors based on interactive skills teaching [31], and was delivered in the context of a high-profile government focus on ‘changing Scotland’s relationship with alcohol’ [32]. Thus the programme reflected many of the ‘lessons learned’ from a similar project in Sweden [33], although both that and other national initiatives have met with modest implementation success [33-35]. In Scotland, ABIs were implemented extensively, with delivery of 470,540 ABIs reported over a six year period, exceeding the target of 332,692 [36]. No previous study has explicitly sought to investigate implementation strategies in this kind of target-driven programme for alcohol.

The aim of this study was to explore experiences of implementation of ABIs in settings outside of primary healthcare in the Scottish national programme in order to identify learning for implementation that may be relevant to any non-primary care setting.

## Methods

### Sample

All eleven local health services (‘boards’) in mainland Scotland (as well as three smaller island NHS boards) initiated programmes of ABI delivery outside of (and within) primary care in response to the national target. In each board, one or more senior practitioners were responsible for their board’s performance in relation to the target (‘implementation leaders’) and for co-ordinating or overseeing related support or service development. We purposively identified experienced individuals from amongst this group, ensuring a range of both high and low performing health boards [37] and those with experience of a variety of settings were interviewed. Representatives of the three island health boards were not included as implementation in that context was judged likely to raise issues specific to their size and context. The final sample size was dictated by the resources available and the timescale for the study and is described in detail in Table 1.

**Table 1:** **Profile of interviewees (n = 14), in line with COREQ and RATS guidelines [** 62,63 **]**

**Job/profession**	**Role e.g. strategic only or clinical (patient-facing) & strategic**	**Health board area**	**In which setting(s), was the interviewee responsible for ABI implementation?**	**Range of ABI delivery performance in the sample for each setting**
1. Alcohol & Drug Partnership (ADP)^a^ Co-Ordinator	Strategic	A	A&E	For each of the 8 health boards for which interviews were conducted, the % of ABIs delivered across primary care, A&E, antenatal and wider settings as a proportion of the total delivered across all settings was available.
			Antenatal	
			‘Wider’^d^ settings	
2. Specialist^b^ Nurse	Strategic	B	A&E	
			Antenatal	
			Wider settings	
3. Specialist Nurse	Clinical & strategic	C	A&E	
4. Senior Medical Doctor^c^ (A&E)	Clinical & strategic	C	A&E	This was used to designate boards as high performing or low performing in each setting as follows:
5. Senior Medical Doctor (Public Health)	Strategic	C	A&E	
			Antenatal	
			Wider settings	
6. ADP Officer	Strategic	D	A&E	High performing = above the median % of overall ABIs delivered in the setting.
			Antenatal	
			Wider settings	
7. Specialist Nurse	Clinical & strategic	D	A&E	Low performing = below or equal to median % of overall ABIs delivered in the setting.
8. Specialist Nurse	Clinical & strategic	D	Antenatal	
9. Senior Medical Doctor (A&E)	Clinical & strategic	E	A&E	These are not indicated specifically for each health board area as it could enable identification of health boards, potentially compromising the anonymity of interviewees.
10. Senior Medical Doctor (Public Health)	Strategic	E	A&E	
			Antenatal	
			Wider settings	
11. Senior NHS Manager	Strategic	E	A&E	
			Antenatal	
			Wider settings	
12. ADP Co-Ordinator	Strategic	F	A&E	
			Antenatal	
			Wider settings	
13. Specialist Nurse	Clinical & strategic	G	A&E	
			Antenatal	
			Wider settings	
14. Specialist Nurse	Strategic	H	Antenatal	
**Totals**				
ADP: 3	Strategic: 8	8 of 11 mainland health board areas	**A&E:** 12	**A&E:** 4 low performing; 4 high performing
Specialist nurse: 6	Clinical & strategic: 6		**Antenatal:** 10	**Antenatal:** 4 low performing; 4 high performing
Senior doctor: 4 (2 public health; 2 A&E)			**Wider settings:** 8	**Wider settings:** 3 low performing; 5 high performing
NHS Manager: 1				

**Duration of Involvement:** Twelve interviewees had been involved in the ABI programme for over five years i.e. at least from the start of the national target, the others for two and three years.

**Non-Participation:** Of those sampled, one individual (a senior midwife) who had initially agreed to take part, failed to respond after several attempts to arrange the interview. No sampled individuals declined to take part.

**Relationship Established:** Prior to interview, five participants were well known, two less well-known and the others not known at all to the interviewer, who was at the time working as an independent researcher.

^a^ADPs are local strategic multi-agency partnerships responsible for taking forward action to address alcohol and drug misuse.

^b^Alcohol/addiction/substance misuse/mental health specialty.

^c^Consultant level.

^d^Wider settings are other settings outside of primary care, including hospital wards, pharmacies, youth and community settings.

### Data collection and ethics

Scottish NHS ethics approval was not required for the study and ethical approval was granted by the Ethics Committee of London School of Hygiene and Tropical Medicine. Interviewees were fully informed about the study by email and followed up by telephone by NF. Full verbal consent was obtained by telephone and audio-recorded also by NF. Semi-structured telephone interviews (averaging 67 minutes in duration) were conducted by NF between September and November 2013. Previous experience interviewing individuals in similar roles found telephone interviews to be preferable as they can be more easily cancelled with little or no notice should urgent clinical commitments arise. Interviewees were provided with a topic guide (Table 2) in advance, which was informed by both ABI-specific and generic implementation literature including the Consolidated Framework for Implementation Research (CFIR) [25]. During interviews, participants were encouraged to speak freely about their experiences, and questions were not asked verbatim of each participant. Particular attention was paid to drawing out participants’ reflections on how best to approach ABI implementation in any new setting outside of primary care. Interviews were audio-recorded, notes simultaneously typed during the interviews and the recordings used afterwards to complete and correct the notes. All notes were subsequently checked for accuracy by interviewees at which point they also had the opportunity to elaborate or clarify any points. In some cases when quoting sections of the notes deemed controversial or sensitive by the participant or the authors, the interview code number is withheld, in order to protect the participant’s anonymity and working relationships.

**Table 2:** **Summary of topic guide**

**Questions from Topic Guide (without prompts under each question)**	
1.	How did you get involved with ABI implementation in X health board?
2.	Who else was involved in the initiative? How were they involved?
3.	When and how were frontline staff involved in the initiative?
4.	How was the delivery of ABI designed to work in this setting?
5.	In your experience, what are the important differences between trying to implement ABI in primary care, and trying to do so in A&E/antenatal care?
6.	How important was the national target and related activities in driving forward implementation?
7.	How sustainable is the delivery of ABIs in your view?
8.	To what extent was implementation influenced by contextual or organisational factors?
9.	From all that you’ve mentioned, what would you pick out as the key lessons for others trying to implement ABI outside of primary care settings?

### Analysis

Analysis was conducted after data collection and familiarisation with checked notes and recordings. As many interviewees had experience of multiple settings and the research question was not specific to these settings, all interviews were analysed together. NF and SH coded segments of interview manually using a simple inductive approach as themes emerged from the interviews. They met to discuss codes and broader themes arising. A framework matrix [38] was used to chart the data, organising the emergent themes into categories including, where relevant, categories relating to the domains of the Consolidated Framework for Implementation Research (CFIR) [25]. The matrix enabled a holistic, descriptive overview of the entire data set to be taken, and for the data to be considered through a broader ‘implementation science’ lens. NF and SH used it to reflect on and refine the overarching themes that were relevant to the research question.

## Results

Five main areas of focus consistently emerged in interviewees’ reports of their experiences of and learning about ABI implementation. Each area is discussed in further detail below and includes interviewees’ views on barriers and helpful strategies for implementation within each one with illustrative quotes. A summary of results is provided in Table 3. Although participants sometimes highlighted elements of culture specific to one setting as a barrier to implementation (e.g. the nature of care in an A&E department), there were few differences in the kinds of helpful strategies identified by interviewees to resolve those barriers. These are outlined below. There were no clear differences in strategies identified in high and low performing health boards in terms of ABI delivery [37]. In line with the research question, the focus here is on those strategies identified as helpful that were common across the settings. Interviewees who reported success in embedding implementation in a particular setting, tended to report that they had used the identified strategies from earlier in their implementation efforts. Many felt that preparation for implementation needed to start early, long before routine or widespread delivery of ABIs was expected. Such a period of preparation would have been helpful, in their view, to get recording and reporting systems in place, to prepare training materials and tailored delivery plans, and to get the support of senior staff and national professional bodies. All interviewees clearly described the challenging nature of ABI implementation.

**Table 3:** **Summary of themes and codes**

**Overarching Areas of Focus**	**Codes**
(1) The national ABI target:	1A: Priority for managers and health boards
	1B: Responsibility for target
	1C: Pressure & distortions
(2) Leadership for implementation:	2A: Senior staff support
	2B: Implementation leader status
	2C: Acknowledging prior effort.
(3) Flexibility, collaboration and pragmatism in intervention design:	3A: Desirable versus possible
	3B: Building on current practice
	3C: Monitoring and crediting pre-existing delivery
(4) Recording, monitoring and reporting:	4A: Early evidence & feedback important
	4B: Difficulty recording
	4C: Mandatory recording
(5) Engaging and supporting frontline staff:	5A: Involving staff throughout
	5B: Training flexibility
	5C: Specialist support both helpful and unhelpful.

### The national ABI target

The target was mostly, but not universally, considered helpful in efforts to secure ABI implementation across all settings. Most felt that the target drew the attention and support of senior managers at health board level, which either started the process of implementation, or increased the priority of ongoing ABI work. Two health boards had made use of local targets since 2012/13 and felt confident that these would ensure continued implementation of ABIs, whether or not ABIs continued to be a focus nationally.

*The target was definitely important. Health boards take the target seriously. They are monitored on them…at the highest level…it probably wouldn’t have happened without it being a target.*

Interview 3, Both setting

Many interviewees felt that there was a lack of clarity locally about whose responsibility it was to ensure that the target was met. This resulted in a lack of ownership: a sense that ‘*everyone thought that it was somebody else’s target’* (interview 2)*.* Only a few health boards subdivided the board target into a specific target for each of the eligible settings: primary care, antenatal, accident and emergency (and later wider settings). As one interviewee put it, this meant that ‘*the lines of accountability were stretched’* (interview 5)*.* Some implementation leaders reported that the target was seen as theirs to deliver, rather than the responsibility of frontline staff in the setting. In addition, the funding provided to support delivery of the target was recognised as being crucial for supporting implementation, as it was used to pay for implementation leaders, training and the adaptation of IT systems (see below).

Some interviewees suggested that the pressure felt at all levels to meet the target had some unintended consequences. These included distortions in recording such that reported ABI figures were felt in some cases to be misleading, delivery of ABIs by staff employed on short-term contracts rather than routine delivery, and resistance from staff who felt ‘*coerced’* (interview 6) into delivering something that was ‘*foisted upon them’* (interview 1)*.*

*People get pressure fatigue and project fatigue…they have multiple demands and multiple targets to meet while also operating under financial constraints with less people to do the work. People get burnout and say ‘I just can’t. I just can’t do any more’ and it’s really difficult to know how to overcome that.*

Interview 1, Both settings

Perceived distortions in recording were often based on differing interpretations of what ‘counted’ as an ABI, in the context of pressure to achieve the target.

*“We have never undertook to provide an ABI and are certainly not doing it or claimed to have done it, but our screening has been interpreted as delivering an ABI”*

Interview number and setting withheld.

Overall, the target was viewed as a ‘*double-edged sword*’ (Interview 7) by some interviewees: important for implementation but creating perverse incentives to maximise reporting of ABI delivery.

### Leadership for implementation

The identification of appropriately senior individuals to lead the implementation process and the support of other senior practitioners and staff from the start of the process, were considered crucial.

*In hindsight if I was running this project again, I would have held that [first] meeting with very senior leads so hospital managers and senior medical leads. I would have rubber-stamped the initiative from very, very high up and I think that might have made things a wee* [colloquial term meaning small] *bit faster perhaps.*

Interview 7, A&E

It was almost universally reported that the process of gaining such senior support was not easy.

*It was very difficult to find a lead, a champion, someone who could grasp and lead it for the department.*

Interview 1, Both settings

Some senior staff were unconvinced of the merits of delivering ABIs in the A&E setting for evidence or appropriateness reasons.

*“One of the concerns is that there’s not actually from our reading of it a strong evidence base for them actually being useful in terms of outcome. There’s a meta-analysis that [a colleague] gave to me [which] suggested that at best there was impact for 3 or possibly 6 months on individual’s patterns of alcohol intake, so the bottom line is that we’ve never been convinced of the evidence base that it’s been a useful thing to do.”*

Interview 11, A&E

Where there was delay in appointing implementation leaders, or where the lines of responsibility for leading and reporting implementation were unclear, interviewees tended to report more of a struggle with the whole process. The status of the implementation leader was important in other ways too. One senior clinician noted that although he and his colleagues did not agree with A&E delivery of ABI, they had co-operated out of respect for the implementation leader with whom they already had an excellent prior relationship.

Some interviewees felt that their efforts were undermined by their own position not being sufficiently senior.

*There’s a pecking order there. It’s harder to move things when you’ve only got responsibility for your own wee team.*

Interview 4, A&E

Many felt that the process of engaging senior staff could be helped by approaching them with an open mind and a readiness to acknowledge existing related work rather than assuming that ABI represented a completely new practice in that setting.

*There was a bit of ‘I’ve got the 10 commandments here, it’s a really good way to live, I don’t understand why you’re not taking them.’ If I had my time again the approach would be different. I would probably have a discussion with senior managers about ‘how we can develop the best practice that you’re delivering at the moment?’*

Interview 1, Both settings

### Flexibility, collaboration and pragmatism in intervention design

Approaching senior managers with an open mind also required a willingness to be flexible and pragmatic about the design of the programme, balancing what was desirable with what was possible.

*A key lesson is not to be afraid to tweak the model. An ABI is a structured conversation. It can be structured around other processes that might make it easier for staff to incorporate in their daily work.*

Interview 3, Both settings

Interviewees noted a need to be pragmatic about what could be delivered in particular settings, particularly A&E, and as a result used minimal screening approaches, or instigated ABIs in other parts of hospital system such as in admissions wards following treatment in A&E.

*We started off with the full FAST screening tool completed on every patient but it was too cumbersome, but now we’ve just gone for the first question and if yes then a conversation about alcohol would be helpful.*

Interview 7, A&E

In antenatal settings, flexibility was felt particularly important in relation to deciding how to screen women by adapting existing forms or processes, ‘*not to invent extra work’* (interview 9) and using appropriate language that ‘*conforms to the language that our midwives would use’* (interview 8)*.*

The process of adaptation was helped by close working relationships with frontline staff as outlined below and a good understanding of current practice. For example, many noted that staff were already asking patients questions about alcohol in both antenatal and A&E settings. Implementation leaders used this knowledge as a starting point for ABI design or to support efforts to ‘*win hearts and minds*’ (interview 14) over to the feasibility and value of ABIs. Some argued that ABIs were already happening but without staff getting credit for their efforts, in an attempt to motivate staff to record and report such delivery.

### Recording, monitoring and reporting

Many interviewees advised that the establishment of practical and robust recording, monitoring and reporting procedures was essential, and that it needed to happen earlier in the implementation process than had occurred. It was suggested that this should have been considered nationally rather than being the responsibility of each health board.

*Unless you can capture delivery then you can’t report it or know about impact. Unless you’ve got recording in place, you can train until the cows come home but you can’t evidence it.*

Interview 10, Both

The ability to provide feedback on delivery figures over time was considered by some to be a crucial tool in the implementation effort.

*We monitored delivery really closely, month to month, and reported back. So if there was a dip one month…when we reported back to the setting, sometimes we would be told that it ‘just slipped off the agenda’ and that they would get back onto it.*

Interview 2, Both

Others cited the value of comparative data:

*If we had robust recording systems then I could say we’re giving you reliable information month by month and…I can see exactly who’s not delivering ABIs…it’s a name and shame game and that’s what you need before anybody is going to buy into it. People say they’re having these conversations now but there’s no evidence for me to go back and say they’re actually delivering the ABIs.*

Interviewee code withheld

This last comment illustrates the fact that in a number of boards, they had not succeeded in putting in place robust recording systems. This was found to be difficult by many interviewees in all settings. In hospital settings, there were multiple points in the care pathway where the ABI process could be initiated or delivered and thus different electronic data capture points potentially operative, and needing to be linked.

*There are four, five, six different systems depending on how patients come in and progress through the NHS system…You cannot expect that because it’s easy to implement a recording and information collection system in one area that it will be easy in another or that you would ask the same people about it.*

Interview 1, Both

Secondly, changing these systems was expensive and generated frustration, partly due to this complexity.

*We’re still in the process of agreeing who has access to [the recording screens] because everybody has access to different levels of the system. So that makes it really difficult trying to negotiate. It shouldn’t be difficult it should be dead easy. You should be able to say every nurse and doctor in A&E should have access to that screen but for some reason it’s not easy. That’s the biggest bane of my life, that’s not easy.*

Interviewee code withheld

Finally, many interviewees reported that it was necessary for recording to be made mandatory or staff simply bypassed the electronic fields.

*When the [IT system] was modified to create the mandatory field, it meant that you couldn’t discharge a patient until you clicked either ‘BI delivered yes/no’ and that was the only thing that significantly increased the recording and now we are well in target.*

Interview 7, A&E

### Engaging and supporting frontline staff

Interviewees emphasised the need to work closely with frontline practitioners during the design, training and implementation stages. The need to ensure that the intervention fitted in with routine practice required input from practitioners.

*Staff were very involved in all decision-making… frontline staff are the best people to tell you what’s going to work and not going to work and that got us a better ‘buy in’ because I think staff felt that they were part of it.*

Interview 3, Both settings

Implementers had to be highly flexible in how they made training available to staff, adapting national training materials to their local context and using them to deliver short courses which staff could realistically attend [3]. The need for flexibility was particularly extreme in the A&E setting and involved trainers coming in very early in the morning, during night shifts, using online learning modules, and/or delivering one to one training on wards. This was very time-consuming.

*We broke [the training] down into bite sizes of one and a half hours… I came in very early so that I could catch the staff starting when they were at their quietest period in the emergency department and each member of staff attended three sessions of one and a half hours. This carried on for ages until everyone was trained – death by a thousand cuts.*

Interview 7, A&E

Many interviewees spoke about the importance of having working relationships on the ground with frontline staff as early as possible in the implementation effort. Indeed those health boards who had implemented ABI delivery projects prior to the announcement of national target were felt to have made much more rapid progress in part because of relationships which were already in place. The role of alcohol liaison or specialist nurses (ALNs) was also seen as helpful in hospitals, both in acting as champions for delivery and supporting staff at the point of ABI delivery.

*I think some of it is about having a closer working relationship with midwives and being more of a presence and support to them. At the moment we are not in and around antenatal wards on a weekly or daily basis. We deliver training and they are on their own. Alcohol Liaison Nurses could offer advice and support to staff with management issues for people at higher risk.*

Interview 3, Both settings

But while seen as desirable by some, this model was not always enough to secure delivery by frontline staff, and having a specialist in the department was reported by others as a way for staff to avoid taking on this role.

*Across the hospital because of the ALNs work, some people know a bit about [alcohol], but the only people having conversations about alcohol [with patients] are the ALNs.*

Interview 2, Both settings

*Part of [the alcohol nurse] role was to involve other staff in what she was doing and to show them how easy it was to do but it probably wasn’t successful in terms of embedding anything into practice because it gave them a get-out.*

Interviewee code withheld

## Discussion

Even with significant national support via a specific delivery target and additional funding, the implementation of alcohol brief interventions in settings outside of primary care proved challenging. The challenges related to competing pressures on staff and the extensive time required to restructure services, train staff in support of the initiative, motivate them to deliver in routine practice, and adapt systems for recording delivery. While implementation leaders identified five broad areas in which to focus implementation efforts, it did not necessarily follow that the implementation strategies they identified as helpful could be successfully employed, and even then implementation was highly time-consuming, and more complex than expected.

Intensive and wide-ranging implementation efforts have previously been found necessary in efforts to systematically change practice in relation to high profile issues in patient care, for example, hand hygiene [39] and HIV testing [40]. In the ABI field, some studies have identified similar themes including support from senior staff [41-44], adapting the intervention to suit current practice [41,43,45,46], and establishing an effective IT system for recording and feedback [43,45]. Others have noted the inadequacy of training to have been a barrier to delivery [41,45,47,48] and advocated a flexible approach similar to that reported by some interviewees in this study.

Much of the literature in this field highlights barriers and facilitators to implementation operating at individual practitioner level such as attitudes or skills or concerns about patient responses to ABIs [49-52]. These factors can be addressed directly [53] but were not explicitly targeted by implementation leaders in this study apart from via practitioner training, perhaps because there was in effect a requirement for ABI delivery. The approaches to implementation identified here as helpful such as targets and senior management support, tended to operate at a system or higher level unlike the factors described in one narrative review of implementation in primary care [27]. A CFIR-informed review also suggested that strategic and organisational factors as well as the process of implementation (rather than practitioner or intervention factors) could be more important for success [54].

The existence of the target for delivery of ABIs was thought to be useful by interviewees, over and above the resources that accompanied it. This kind of top-down performance management in the NHS can generate pressure on staff, focus attention on specific issues rather than holistic care, and may not always result in the intended change [55-57]. Similar concerns arose in this study; nevertheless, the few reports of distortions in the reported ABI figures seem to result from two main issues: firstly, a sense that the programme merely recorded rather than changed prior practice; and secondly conflicting views of what constitutes an ABI and ‘should’ therefore ‘count’ towards the target. The target was seen by interviewees as helpful on balance, though this finding has arisen in the specific context of Scotland’s national policy approach to addressing alcohol-related harm.

The need to adapt the intervention to fit with current practice in each setting in order to make it easier and more acceptable to implement, was also highlighted as important in the Swedish national ‘risk drinking project [33]. However, it carries a risk of compromising the effectiveness of the intervention in ways that are currently unknown, due to the limited extent of rigorous effectiveness study in this area, and this was reflected in the uncertainty of interviewees as to what actually constituted an ABI. Interventions in the substance misuse field need to be clearer about core and adaptable components [58], and the lack of study of the required content of ABI for efficacious and effective delivery is a recognised weakness of the existing literature [3,59,60].

The absence of a preparation period prior to the national roll-out of the target did not allow for development or intensive piloting of interventions. Given the weaknesses in the evidence base for ABIs, and the level of resourcing of the national programme, it could be seen as a missed opportunity that evaluation of the initiative did not include effectiveness studies [30]. The lack of both stronger evidence of ABI effectiveness in settings outside of primary care and earlier piloting and practical adjustments to ABIs including recording systems and associated training specific to each setting, was problematic for implementation in some areas in this study. Addressing these issues should be expected to be helpful for gaining the support of senior staff in similar initiatives in future [61].

This study captured the views of very experienced ABI implementation leaders involved in a uniquely high-profile and ambitious national implementation programme. Their reports are specific to this context in Scotland, and may not be transferable to other areas. The retrospective qualitative approach taken could not provide empirical evidence for the implementation strategies advocated by interviewees. A greater sample size may also have enabled a more in-depth comparison between different settings and perspectives, and may have enabled data saturation to be achieved. Interviewees varied however, in terms of setting, role and the size and demography of their health board area. As such their accounts are striking in their similarity, more so than in their diversity. Eight of the 14 had experience of implementation in more than one setting, making them well placed to suggest what might be important across settings. The identified factors affecting implementation also fit well however with issues known to be important in implementation efforts more broadly as represented in the CFIR [25]. Future national or large-scale ABI programmes could be utilised as opportunities to explore the importance of the five strategies identified here and identify other useful strategies, notwithstanding the perhaps more urgent need for further research into efficacy, effectiveness and intervention design [59,60].

## Conclusions

The participants in this study were responsible for the delivery of a high profile national programme that was supported by considerable resources and funding. The implementation of the programme proved complex and challenging in many respects. These implementation leaders did not benefit from prior knowledge of, or training in, implementation science. The learning reported by them was therefore in many cases gained ‘the hard way’ through trial and error, when many issues could have been anticipated in advance. This interview study emphasises the magnitude of the challenges involved in large scale ABI implementation efforts, as well as identifying useful learning from implementation leaders.

## Abbreviations

ABI: Alcohol brief intervention
A&E: Accident and emergency
CFIR: Consolidated framework for implementation research
